# Preparation of Amino-Functionalized Poly(*N*-isopropylacrylamide)-Based Microgel Particles

**DOI:** 10.3390/gels9090692

**Published:** 2023-08-28

**Authors:** Anna Harsányi, Attila Kardos, Imre Varga

**Affiliations:** 1 Institute of Chemistry, Eötvös Loránd University, Pázmány Péter sétány 1/A, H-1117 Budapest, Hungarykardosa@ujs.sk (A.K.); 2 Department of Chemistry, J. Selye University, 945 01 Komárno, Slovakia

**Keywords:** responsive microgels, cationic microgels, primary amine functionalized microgel, EDC coupling

## Abstract

Responsive cationic microgels are a promising building block in several diagnostic and therapeutic applications, like transfection and RNA or enzyme packaging. Although the direct synthesis of cationic poly(*N*-isopropylacrylamide) (PNIPAm) microgel particles has a long history, these procedures typically resulted in low yield, low incorporation of the cationic comonomer, increased polydispersity, and pure size control. In this study, we investigated the possibility of the post-polymerization modification of P(NIPAm-co-acrylic acid) microgels to prepare primary amine functionalized microgels. To achieve this goal, we used 1-ethyl-3-(3-(dimethylamino)propyl)carbodiimide hydrochloride (EDC) mediated coupling of a diamine to the carboxyl groups. We found that by controlling the EDC excess in the reaction mixture, the amine functionalization of the carboxyl functionalized microgel could be varied and as much as 6–7 mol% amine content could be incorporated into the microgels. Importantly, the reaction was conducted at room temperature in an aqueous medium and it was found to be time efficient, making it a practical and convenient approach for synthesizing primary amine functionalized PNIPAm microgel particles.

## 1. Introduction

Responsive polymer gels are solvent swollen polymer networks, whose swelling shows a large, non-linear, reversible change in the function of external physical or chemical characteristics of their environment. Examples of stimuli that can trigger the responsive behavior of gels include temperature [1], pH [2], magnetic fields [3], ionic strength [4], and light [5]. These gels have already found applications in various fields such as biosensing [6], drug delivery [7], microreactors [8], and catalysis [9].

One of the most widely investigated responsive gels is poly(*N*-isopropylacrylamide) (PNIPAm). PNIPAm has a lower critical solution temperature (LCST). Below this temperature (32 °C), the polymer is completely water-soluble, but above the LCST phase separation occurs. Therefore, PNIPAm gels collapse above this temperature, and they lose most of their water content resulting in about an order of magnitude reversible volume change in a narrow temperature range [10,11]. This phenomenon is usually referred to as the volume phase transition of the gel (VPT). An important feature of PNIPAm is that its LCST facilitates the preparation of practically monodispersed responsive microgel particles by means of precipitation polymerization. In a typical batch polymerization of the PNIPAm microgel particles, the degassed aqueous solution of *N*-isopropyl acrylamide (NIPAm) and *N*,*N*′-methylene-bis-acrylamide (BA, cross-linker), as well as any comonomer and surfactant, is heated to 60–80 °C, and then the polymerization reaction is initiated by the addition of an initiator. Generally used initiators are anionic persulfate molecules or cationic azo-compounds [12,13]. After the addition of the initiator, radicals are formed, and the polymer chains rapidly grow in the solution until they reach a critical length, whereupon they become insoluble in water and hence collapse. The collapsed polymer chains lack colloidal stability under the reaction conditions, so their aggregation takes place. The aggregation continues until the precursor particles gain enough surface charge to gain colloidal stability due to the accumulation of the charged initiator fragments in the aggregates or the adsorption of ionic surfactants added to the reaction mixture. The polymerization reaction and the growth of the stabilized precursor particles continue until monomers are present in the reaction media. Generally, it is considered that the locus of the polymerization is on the outer surface of the precursor particles [1,14]. However, recently Virtanen et al. proposed an alternative mechanism, whereby the main locus of the polymerization is in the continuous phase of the reaction mixture, from where the collapsed polymer globules diffuse to the existing particles [15,16,17]. Importantly, the size of the prepared microgel particles can be controlled in a wide range (e.g., from ~30 nm to ~1 micron) by the concentration of the ionic surfactant added to the reaction mixture [18,19].

While the classical batch precipitation polymerization produces practically monodispersed PNIPAm microgel particles, these microgel beads do not have a homogenous internal structure due to the faster polymerization of the crosslinker molecules (BA) [20], which leads to the formation of microgel particles with a highly crosslinked inner core and a sparsely crosslinked shell [21,22,23]. Later, it was shown by Acciaro et al. that by keeping the concentrations, and more importantly the concentration ratio of NIPAm and BA, constant during the synthesis by feeding monomers into the reaction mixture, homogeneously crosslinked PNIPAm particles can be synthetized [24].

To enhance the functionalities of PNIPAm-based microgel particles, several studies explored the copolymerization of *N*-isopropylacrylamide with either ionic or non-ionic comonomers [25,26,27,28,29]. One of the important limitations to the quantity and quality of the applied comonomer is that the growing polymer chains must lose their water solubility under the reaction conditions to facilitate the precipitation polymerization mechanism. For instance, it was found that the acrylic acid content in the microgel is limited to ~15 mol%, since using more acrylic acid hinders the collapse of the oligomers and prevents the formation of crosslinked PNIPAm microgels in the reaction mixture [1,30].

Munier et al. obtained similar results to Snowden et al. when they investigated the copolymerization of 2-aminoethylmethacrylate (AEM) into PNIPAm microgel particles to prepare primary amin functionalized microgels [31]. They found that when the AEM concentration was increased above 0.5% of the total monomer concentration, the water-soluble oligomer polyelectrolyte fraction of the reaction mixture that had not been incorporated into the microgel particles started to steeply increase and when more than 1 mol% AEM was applied the microgel particles became rather polydisperse. Similar results were found both in the case of the copolymerization of dimethylaminoethyl methacrylate (DMAEMA) [32,33] and *N*-(3-aminopropyl) methacrylamide hydrochloride (APMH) [34]. Very recently, Annegarn et al. [35] made a systematic investigation to characterize if the increasing pH and the consequent deprotonation of the APMH repeat units in the water-soluble oligomer chains could counterbalance the negative effects of APMH copolymerization into the microgel particles. They found that by increasing the reaction pH to 9.5, the yield of the microgel production indeed increased to 47 w% from its original value of 24 w% at pH = 2.6, and this was accompanied by the increasing copolymerization of the APMH monomer into the microgel particles (74 mol% vs. 31 mol%). However, when the pH was further increased the gel phase precipitated from the reaction mixture due to the loss of the colloidal stability of the growing microgel particles, thus underlying the need for precise pH control during the reaction.

Altogether, it can be concluded that the efficient direct synthesis of the primary amine containing PNIPAm microgels still remains a challenging task, most importantly due to the poor yield of the microgel production and the low incorporation of the cationic monomer in the microgel particles. 

A potential alternative to the direct synthesis of functionalized PNIPAm microgels is the application of post-polymerization modification reactions to introduce the required functional moieties into the microgel particles. For example, Meng et al. prepared both carboxylic and azido group as well as carboxylic and alkynyl group functionalized pNIPAm microgels [36]. Subsequently, they used copper(I)-catalyzed azide-alkyne 1,3-dipolar cycloaddition to functionalize the azido or the alkynyl groups in the microgels, and then in a separate reaction, they modified the carboxylic groups using the 1-ethyl-3-(3-(dimethylamino)propyl)carbodiimide hydrochloride (EDC) coupling reaction. The specificity of these coupling reactions was also thoroughly demonstrated. In another experiment, Wei et al. prepared P(NIPAm-co-AAc) microgel particles. The AAc groups of these microgel particles were modified using 4′-amino-benzo-12-crown-4-acrylamide (B12C4) through the EDC-mediated coupling reaction. This post-polymerization modification allowed the introduction of molecular recognition receptors into the microgel particles [37].

The post-polymerization conversion of functional groups in copolymer microgel particles is also a feasible approach for the incorporation of primary amine functionalities into the PNIPAm microgel particles. To achieve this goal, Shiroya et al. prepared acrylamide (AAm) containing P(NIPAm-co-AAm) copolymer microgels, and then they performed a Hofmann reaction by reacting the microgel particles with a sodium hydroxide/sodium hypochlorite mixture for 1 h at 4 °C [38]. Horecha et al. [39] used a similar approach. They also prepared acrylamide containing copolymer P(NIPAm-co-AAm) microgel particles. The AAm repeat units were converted into vinylamine (VAm) via Hofmann rearrangements by treating the microgel particles with diacetoxyiodobenzene (DIAB). Although primary amino groups were successfully introduced into the microgel-network, the efficiency of these methods has not been addressed.

Another possible method to insert primary amine groups into PNIPAm microgels is the usage of protected monomers, where the amino part of the monomer is capped with a protective side group. The protected monomer is added to the reaction mixture at the beginning of the polymerization reaction. After the completion of the polymerization, the protective side group can be removed to obtain the amine containing microgels. Singh et al. used fluorenyl-methoxy-carbonyl-protected polyethylene glycol methacrylamide (Fmoc-PEG-APMA) macromonomer to prepare P(NIPAm-co-Fmoc-Peg-APMA) microgel. After the reaction, the protective group (Fmoc) was removed by treating the microgels with 20% piperidine at room temperature for 20 min. The microgels were purified by dialysis against water for two weeks. Although Singh et al. have successfully introduced primary amin groups into the microgel, the method is very time consuming, and the protected monomers are hardly available [40].

EDC coupling is a powerful tool to couple primary amines to carboxylic groups in aqueous media. It is often used to immobilize biomacromolecules on various supports or for antibody labeling with fluorophores. Since carboxyl functionalities can be easily and efficiently introduced into PNIPAm microgels by the copolymerization of acrylic acid (AAc) [41,42], EDC coupling is a promising approach to introduce specific functionalities into P(NIPAm-co-AAc) microgels. This approach has already been successfully applied to functionalize P(NIPAm-co-AAc) microgels with fluorescent dyes [36] and proteins [43,44,45], as well as with a crown ether [37]. In this work, we investigate if EDC coupling is suitable for the efficient transformation of the carboxyl groups of P(NIPAm-co-AAc) microgels into primary amine groups using a diamine as a reagent. To achieve this goal, first we prepared PNIPAm microgel particles with 10 mol% acrylic acid content, and then in a second step, the carboxyl groups within the microgel particles were activated using *N*-ethyl-*N*′-(3-(dimethylamino)propyl)carbodiimide (EDC) in the presence of a diamine (cadaverine). The products of the reactions were characterized and the carboxyl/EDC ratio as well as the reaction time were optimized to achieve maximum conversion. Importantly, the reaction was conducted at room temperature in an aqueous medium and it was found time-efficient, making it a practical and convenient approach for synthesizing primary amine functionalized PNIPAm microgel particles.

## 2. Results and Discussion

### 2.1. Synthesis and Characterization of P(NIPAm-co-10%AAc) Microgels

**Figure 1:**
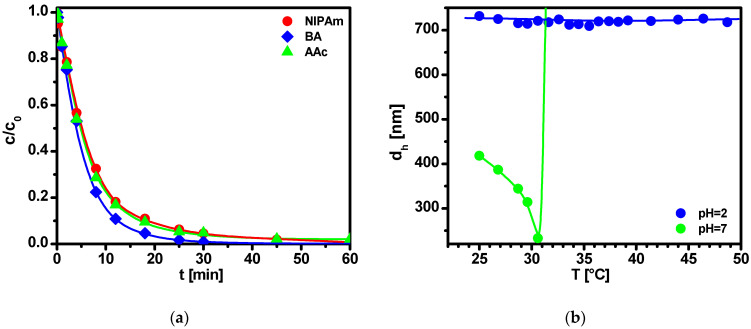
(**a**) The relative monomer concentration in the function of the polymerization time for the P(NIPAm-co-10%AAc) synthesis. (**b**) The hydrodynamic diameter of the purified P(NIPAm-co-10%AAc) microgels in 10 mM HCl (pH = 2) and in 10 mM phosphate buffer (pH = 7) as a function of temperature. Each data point is the average of at least ten measurements and the standard errors of the datapoints are commensurate to the sizes of the symbols in the figure. The PDI of the samples were below 0.1.

In order to prepare primary amine functionalized PNIPAm microgels, first we synthesized PNIPAm microgels with 10 mol% acrylic acid content using batch precipitation polymerization. The prepared microgel particles had an average crosslink density of *R* = 70 (where *R* = *n_NIPA_*/*n_BA_*) and 10 mol% nominal acrylic acid content. To follow the formation of the microgel particles, samples were regularly taken from the reaction mixture and the monomer concentration was determined in the samples by HPLC. As shown by Figure 1a, practically all monomers react within 60 min. In agreement with the literature results, the crosslinker monomer (BA) reacts faster than the other two monomers [19,20,21,22]. At the same time, NIPAm and AAc are consumed at practically the same rate indicating that the acrylic acid is distributed uniformly within the gel network. The prepared microgel was purified by repeated centrifugation and redispersion, and finally it was freeze-dried. The yield of the microgel synthesis was 81%, which is in good agreement with the previous literature results [18,46,47]. Finally, the acrylic acid content of the purified microgel was determined by acid base conductometric titration, which indicated that 84% of the acrylic acid feed built into the microgel particles. This is in good agreement with the yield of the microgel synthesis and the monomer conversion results that indicated the uniform incorporation of acrylic acid into the microgel network.

To demonstrate the effect of the 10 mol% ionizable repeat unit on the swelling and on the VPT temperature of the microgel particles, we measured the hydrodynamic diameter of the purified microgel in the function of temperature at pH 7 (in 10 mM phosphate buffer, where the acrylic acid groups are fully charged) and at pH 2 (in 10 mM HCl, where the acrylic acid moieties are protonated and thus uncharged). The results of these measurements are plotted in Figure 1b. At pH = 7, the microgel particles are highly swollen due to the high charge density of the polymer network. As is evidenced by the figure, with increasing temperature the hydrodynamic diameter of the charged microgel particles remains practically constant in the entire investigated temperature range (from 25 to 50 °C). This clearly indicates that the 10 mol% charged repeat unit content of the PNIPAm chains is sufficient to suppress the collapse of the PNIPAm chains and its VPT is increased far above the investigated temperature range [48]. At the same time, the swollen and uncharged microgel particles at pH = 2 are much smaller and their collapse occurs at ~32 °C as expected. However, in this case the collapsed microgel particles lose their colloidal stability and aggregate in the lack of sufficient surface charge. 

### 2.2. Post-Polymerization Modification of the P(NIPAm-co-10%AAc) Microgels to Primary Amine Functionalized Microgels by EDC Coupling

**Scheme 1:**
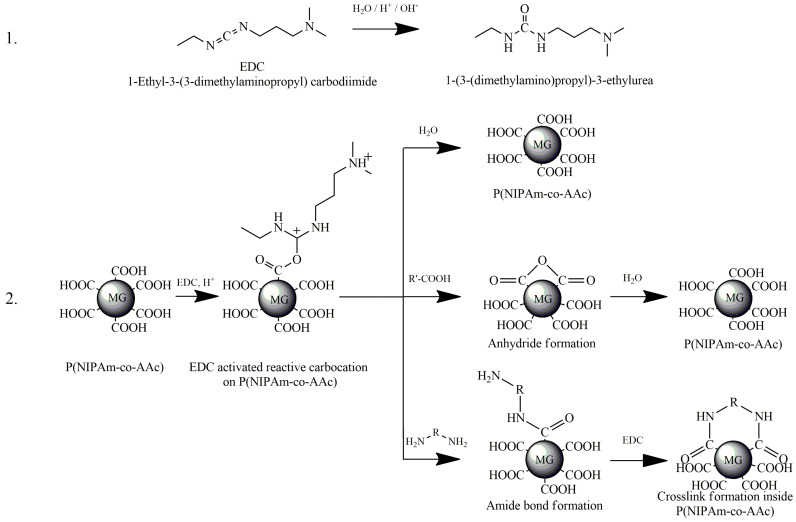
The main reactions taking place and the main products in an aqueous EDC solution in the presence of carboxyl functionalized microgels. **1**. The acid and base catalyzed hydrolysis of EDC. **2**. The EDC activation of the microgl carboxyl groups and three main reactions of the O-acylisourea intermediate ester. EDC activated reactive carbocations can undergo several other potential side reactions which are not depicted here. For additional information see Ref. [51].

EDC is a water soluble carbodiimide, which readily reacts with carboxylic groups to produce an O-acylisourea intermediate ester. The intermediate is activated by H^+^ binding, which yields a reactive carbocation (Scheme 1). This activated carbocation readily reacts with primary amines at room temperature to form an amide and a urea byproduct. At the same time, it can also react with water molecules to regenerate the initial carboxylic group or with another carboxyl group to produce an anhydride, which in turn can regenerate the initial carboxyl groups after their hydrolysis [41]. The reaction scheme is further complicated by the direct hydrolysis of EDC in water, which is efficiently catalyzed by acids and alkali. According to the literature, to minimize the direct hydrolysis of EDC, the coupling reaction is typically achieved between pH 5 and 7 [49,50]. Since EDC could also react with phosphate groups [51], usually a MES buffer is recommended to set the pH. To follow this protocol, we performed the coupling reaction in a 50 mM MES buffer at pH = 5.5. 

To incorporate a primary amine functionality in the microgel, a diamine had to be coupled to the activated carboxyl group (Scheme 1). Nonetheless, a pending primary amine group that has already been coupled to the microgel could also react with another EDC functionalized carboxyl group establishing a new crosslink within the microgel particle and resulting in the loss of the amine functionality. To minimize this side reaction, we used the diamine in an order of magnitude excess in the reaction mixture in every case. Finally, it should be noted that since short chain alkane diamines are typically highly toxic [52], we decided to use cadaverine (pentane diamine) that has only a low toxicity [53], but it still has sufficient solubility in water so it is easy to work with.

**Figure 2:**
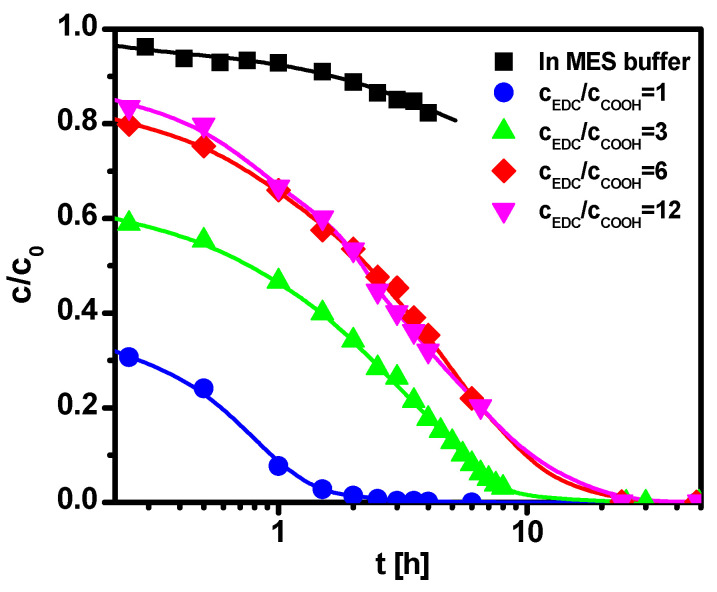
The relative concentration of EDC compared to its initial concentration as a function of reaction time in pure 50 mM MES buffer (black square). All other datasets were measured in EDC/P(NIPAm-co-10%AAc) solutions with a constant 5.0 mM acrylic acid content. Blue circles: stoichiometric EDC/carboxyl group ratio; Green up triangles: 3-fold, red diamonds: 6-fold, and magenta down triangles: 12-fold EDC excess. All samples contained 50 mM MES buffer and had a pH of 5.5.

To optimize the functionalization reaction, we investigated the kinetics of the microgel activation by EDC and then we explored how large EDC excess is required to maximize the cadaverine coupling to the microgel. To gain information about the activation kinetics, we prepared microgel/EDC mixtures with 5 mM acrylic acid content and with five different EDC concentrations. In the first case, we used a stoichiometric amount of EDC compared to the carboxylic acid concentration, and then we increased the EDC concentration to 3, 6, 12, and 24-fold excess. To follow the coupling kinetics, we took samples regularly in the first 6–8 h, and then samples were taken after 24- and 48-h reaction time. It should be noted that in the case of 24-fold EDC excess, samples were taken only after 24- and 48-h reaction time. The EDC content of the samples was determined by HPLC, and the results of these measurements are plotted in Figure 2 on a relative scale compared to the initial EDC concentration. As a reference, we also followed the decomposition of EDC (5 mM) in a 50 mM MES buffer. These control measurements clearly show that the EDC hydrolysis is much slower in the pure buffer than in the presence of the microgel, so the depletion of EDC from the reaction mixture is mostly related to its reaction with the acrylic acid functionalized microgel. When increasing the initial EDC concentration, the reaction of EDC seems to approach a limiting curve, but after 24 h each reaction mixture was completely depleted in EDC. Thus, we made 24 h coupling reactions in all further experiments.

As a next step, we also introduced cadaverine into all reaction mixtures in 10-fold excess (50 mM). After 24 h reaction time, the functionalized microgels were purified by ultrafiltration using Amicon Ultracentrifugal Filters (MWCO = 30 kDa) and the hydrodynamic diameter of the functionalized particles was measured by dynamic light scattering. Unfortunately, these measurements revealed that regardless of the applied EDC excess, a significant interparticle coupling of the individual microgel particles took place during the reaction. To suppress this interparticle coupling reaction, we repeated the cadaverine coupling using a more dilute microgel solution (at 1 mM carboxyl concentration), while otherwise using the same reaction conditions (10-fold, 10 mM, cadaverine excess, 50 mM MES buffer, and 1 through 24-fold EDC excess). The functionalized microgels were purified by ultrafiltration using the same Amicon Ultracentrifugal Filters as previously described. In this case, dynamic light scattering revealed only a small amount of crosslinking of the microgel particles that could be easily removed by filtering the samples with a 0.8 µm membrane filter. To characterize the effect of EDC excess on the efficiency of the cadaverine coupling, we measured the electrophoretic mobility of the cadaverine functionalized microgels as the function of solution pH.

**Figure 3:**
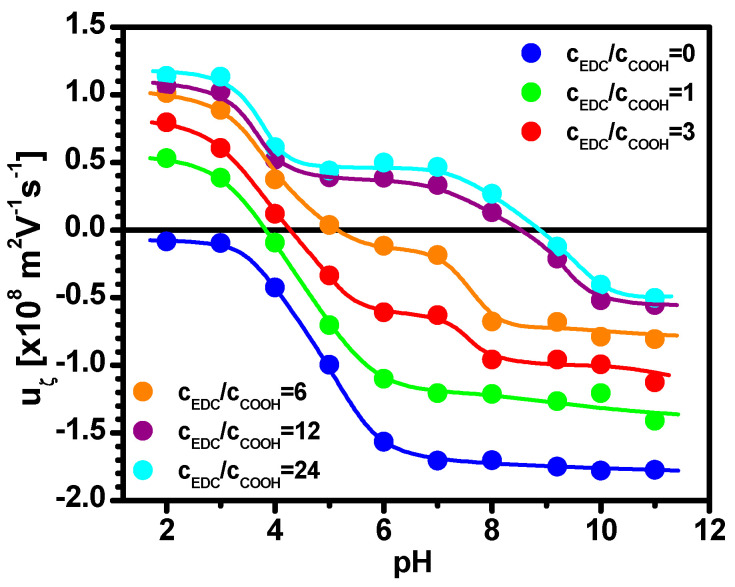
The electrophoretic mobility of the functionalized microgel particles as a function of solution pH. Different colors indicate microgels functionalized in different excesses of EDC as defined by the legends in the figure.

As is shown in Figure 3, the electrophoretic mobility of the unfunctionalized P(NIPAm-co-10%AAc) microgel never becomes positive regardless of the solution pH. At low pH values, the electrophoretic mobility of this microgel is practically zero but with increasing pH, as the carboxylic groups become deprotonated (pK_a_~5), the electrophoretic mobility of the microgel quickly increases, and it reaches a large negative value above pH 6. When the microgels are reacted with cadaverine in the presence of a stoichiometric amount of EDC, the electrophoretic mobility curve is shifted to the positive direction. At low pH, where both the carboxyl and amine groups are protonated, the microgel becomes positive indicating the incorporation of some primary amine groups into the microgel. However, as the pH increases the microgel quickly becomes negatively charged showing the significant excess of the carboxyl groups compared to the amount of the grafted primer amine groups. As the amount of EDC is increased in the reaction mixture, the mobility curve is significantly shifted towards the positive values up to 12-fold EDC excess. However, a further increase to 24-fold EDC excess results only in a small increase in the mobility values, implying that the primary amine content of the microgel cannot be further increased efficiently by further increasing the EDC concentration in the reaction mixture. 

From 3-fold EDC excess, a well-defined mobility plateau develops between pH 4 and 8 in the mobility curve. In this pH range, both the amine and the carboxyl groups are charged in the microgel particles, and thus the sign of the electrophoretic mobility in this pH range reveals which functional group is present in excess in the microgel. As is shown in the figure, when the reaction is performed in 6-fold EDC excess the microgel has a close to zero mobility in this pH range, indicating that it contains carboxylic and amine groups in roughly equal amounts. When the EDC excess is increased further in the reaction mixture, the microgel becomes positive in this intermediate pH range revealing the excess of the primary amine groups in the microgel. At the same time at high pH values, where the amine groups become deprotonated and uncharged, the electrophoretic mobility becomes once again negative. This clearly shows that not all carboxyl groups become functionalized in the microgel particles.

**Figure 4:**
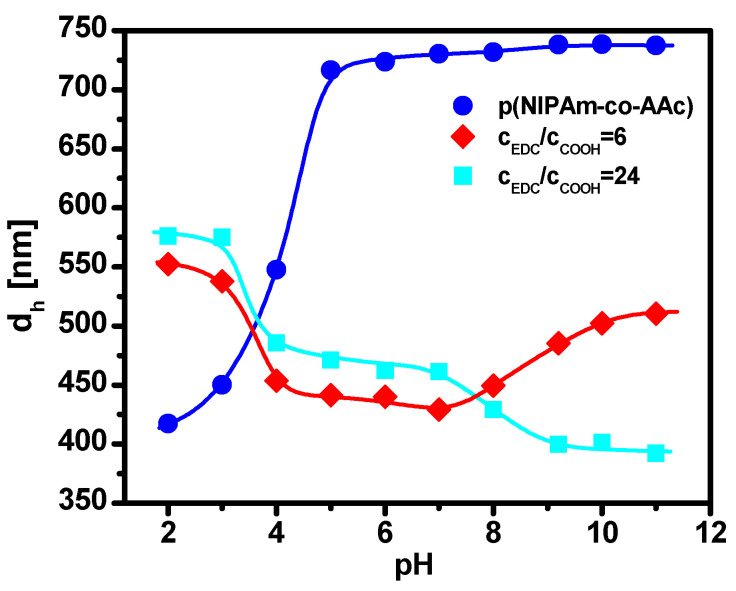
The hydrodynamic diameter of the P(NIPAM-co-10%AAc) (blue circle) and two of the amine functionalized microgels as a function of solution pH. Red diamonds indicate the microgel functionalized in 6-fold EDC excess, while light blue squares represent the microgel functionalized in 24-fold EDC excess. Each data point is the average of at least ten measurements and the standard errors of the datapoints are commensurate to the sizes of the symbols in the figure. The PDI of the samples were below 0.1.

To further characterize the amine functionalized microgels, we measured their hydrodynamic diameter in the function of solution pH. For the sake of simplicity, only three of these curves are plotted in Figure 4. Considering that the swelling of the microgel particles varies with their overall charge density, the pH dependent swelling of the microgels is in good agreement with the previous electrophoretic mobility results. The swelling of the non-functionalized microgel steeply increases as the carboxyl groups become charged with increasing pH. At the same time, the swelling of the other two microgels decreases in this low pH range since the increasing negative charge of the carboxyl groups compensates some of the positive charges provided by the protonated amine groups. It should be noted that the electrophoretic mobility measurements implied roughly equal amounts of carboxyl and amine groups for the sample functionalized in 6-fold EDC excess. Thus, in the pH range where both the carboxyl and the amine groups are charged, this microgel has a minimum swelling and as the pH is increased further (pH > 7) the microgel reswells due to the deprotonation of the amine groups, which gives rise to an increasing excess of the negative carboxyl groups and the increasing overall charge density of the microgel. The situation is different at high pH for the microgel functionalized in 24-fold EDC excess. In this case, above pH ~ 7 the swelling of the microgel further decreases. This reflects that the amine groups are in significant excess compared to the carboxyl groups in this sample, since the deprotonation of amines results in the decreasing charge density of the microgel despite the presence of some carboxyl groups.

**Figure 5:**
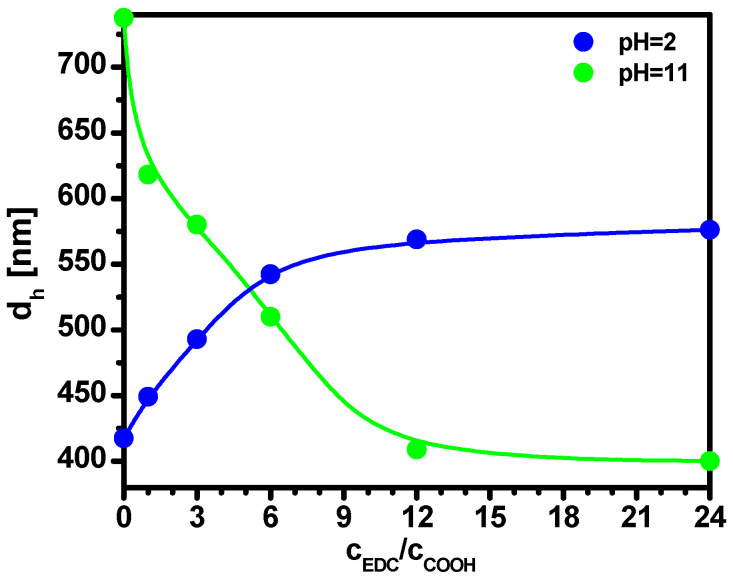
The hydrodynamic diameter of the amine functionalized microgels measured at pH = 2 (positively charged microgels) and at pH = 11 (negatively charged microgels) as a function EDC excess used in the coupling reaction. Each data point is the average of at least ten measurements and the standard errors of the datapoints are commensurate to the sizes of the symbols in the figure.

Since the swelling of the microgels is highly sensitive to the overall charge density of the polymer network, we also plotted the hydrodynamic diameter of the functionalized microgels as the function of the EDC excess used in the coupling reaction (Figure 5). In this case, the hydrodynamic diameter is plotted at pH = 2 and 11, since in the former case only the amine groups are charged in the microgel and in the latter case only the carboxyl groups. As expected at pH = 11, the swelling of the microgels steeply decreases with the increasing amount of EDC used in the coupling reaction due to the decreasing amount of unreacted carboxyl groups remaining in the microgel particles after the coupling reaction. At the same time, the swelling increases at pH = 2 due to the increasing amount of the primary amin groups grafted to the microgel. It should be noted that significant change in the microgel swelling is caused by the increasing EDC concentration of the reaction mixture only before 12-fold EDC excess is reached. This implies that the further increase of the EDC concentration in the reaction mixture causes only a slight increase in the amount of carboxyl groups converted to primary amines. It is also interesting to note that the microgel prepared in 6-fold EDC excess shows similar swelling at pH 2 and 11, implying close to identical amounts of carboxyl and amine groups in this sample. This is in good agreement with the previous electrophoretic mobility results. At the same time, the swelling change between the amine functionalized microgels prepared in 6 and 12-fold EDC excess is significantly different at pH 2 and 11. In the former case, only a slight increase is observed (from 540 to 570 nm), while in the latter case a large decrease (from 510 to 410 nm) happens. In addition, the maximum swelling of the positively charged amine functionalized microgel (575 nm; pH = 2, 24-fold EDC excess) is much smaller than the maximum swelling of the carboxyl functionalized negatively charged microgel (740 nm; pH = 11, no EDC coupling). These observations may be related to three factors. On the one hand, the positive charge density of the functionalized microgels is smaller than the negative charge density of the unmodified microgel, since as is implied by the mobility data, some of the carboxyl groups could not be converted to primary amine groups. Furthermore, a fraction of the grafted diamines may react further and act as a crosslinker, thus limiting the swelling of the microgel. Additionally, the grafted cadaverine molecules introduce short (*n_C_* = 5) alkyl chains into the microgel network which increases the hydrophobicity of the polymer network, and thus it can also decrease the overall swelling of the microgels. 

To determine how much carboxyl and how much primary amine groups are present in the microgel functionalized in the presence of 24-fold EDC excess by cadaverine, we utilized a method based on the interaction of polyelectrolytes (PE) with oppositely charged surfactants. As is well-established in the literature [54], when an oppositely charged surfactant is added to a polyelectrolyte, a large fraction of the surfactant can bind to the polyelectrolyte forming micelle-like surfactant aggregates wrapped by the polyelectrolyte chain, thus decreasing the overall charge of the PE-S complex with increasing surfactant binding. As proposed by Meszaros et al., this can be utilized to determine the amount of PE-bound surfactant by electrophoretic mobility measurements (for details see Ref. [55]). To determine the carboxylic acid content of the microgel, we set the pH of the sample to 12, and thus due to the deprotonation of the amine groups the microgel particles contained only negatively charged groups. We investigated the binding of cetyltrimethylammonium bromide (CTAB) on this negatively charged microgel to determine its acrylic acid content. To determine the amine content of the same microgel sample, the pH was set to 2, and thus the microgel contained only the primary amine groups in charged form. We then investigated the binding of sodium dodecyl sulfate on this positively charged microgel to determine the amount of primary amine groups present in the microgel. As a reference, we also determined the amount carboxyl and amine groups in the P(NIPAm-co-10%AAc) microgel with the same method. 

The results of the binding measurement are summarized in Table 1. As is indicated by the data, about two thirds of the carboxylic groups are converted to primary amine groups in the most efficient functionalization reaction (made in the presence of 24-fold EDC excess). This is sufficient to introduce ~6.3 mol% amine functionality into the microgel network, while ~3.5 mol% of the repeat units remains acrylic acid. We hypothesize that the underlying reason for the suppression of further functionalization of the remaining carboxylic groups is that the grafted amine moieties are positively charged at the reaction pH, and thus when the grafted amine groups become in excess compared to the carboxyl groups, the positively charged microgel macroion becomes depleted in the similarly charged cations including the positively charged cadaverine, and thus the coupling reaction slows and the hydrolysis of the active ester becomes the dominant process.

**Figure 6:**
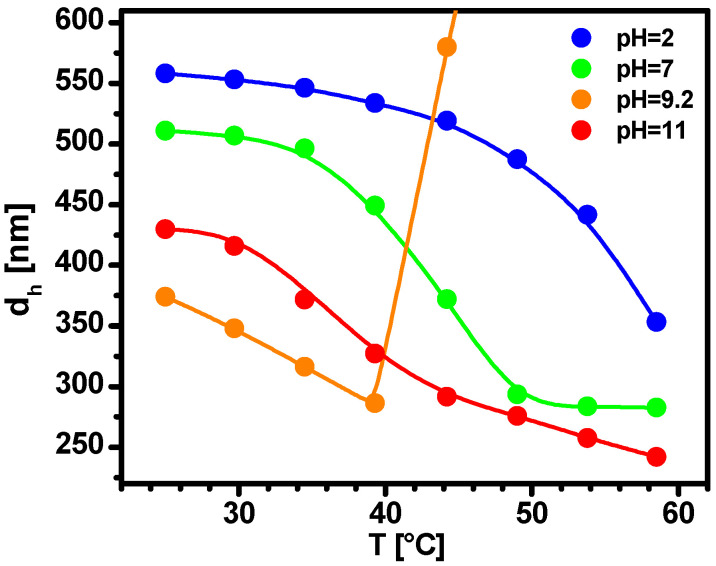
The hydrodynamic diameter of the amine functionalized microgel (made in 24-fold EDC excess) in the function of temperature at four different solution pH values. Each data point is the average of at least ten measurements and the standard errors of the datapoints are commensurate to the sizes of the symbols in the figure.

Finally, we investigated the effect of the amine functionalization on the volume phase transition temperature of the microgel. In this study, we used the microgel sample that contained the most primary amines (made in 24-fold EDC excess) and measured the microgel swelling from 25 to 60 °C at four different pH values (see Figure 6). At pH = 2, the shrinking of the microgel with increasing temperature can be clearly observed above 40 °C, however, the volume phase transition temperature (VPTT) of the microgel has not been reached until 60 °C. This clearly shows that enough amine groups are grafted to the polymer network to turn it hydrophilic in the entire investigated temperature range. At pH = 7, where the acrylic acid repeat units also become charged, the amine groups thus become partially charge-neutralized as the swelling of the microgel particles decreases and a well-defined collapse temperature can be identified at ~50 °C. When the pH is further increased to 9.2, the amine groups become partially deprotonated and the microgel particles lose their overall charge. This results in the further shrinking of the microgel and at ~40 °C the microgel particles lose their colloidal stability in the lack of sufficient surface charge density and they aggregate. This aggregation interferes with the determination of VPTT in this case, yet nonetheless it is evidently much higher than that of the PNIPAm microgel (32 °C). This shows that even if the charges of the carboxyl and amine groups compensate each other within the microgel network, their presence makes the polymer chain more hydrophilic than the PNIPAm chain resulting in an increased LCST of the polymer chain. However, the loss of the excess charge of the microgel leads to the loss of the colloidal stability of the collapsed microgel particles. Thus, when the pH is further increased to 11, where the microgel has a net negative charge, the microgel regains its colloidal stability in the entire temperature range. Interestingly, the microgel shows a two-stages deswelling at this pH, which is usually characteristic for core-shell microgels with a charged shell [56]. This may hint that the unmodified acrylic acid groups are mainly accumulated in the outer shell of the microgel particles. 

## 3. Conclusions

The post-polymerization modification of PNIPAm microgels functionalized with carboxyl groups was found to be an efficient and straightforward method to prepare primary amine functionalized microgels. The main advantage of the presented approach is that while the direct synthesis of amine functionalized PNIPAm microgels leads to low yield, low incorporation of the cationic comonomer into the microgel particles, and increased microgel polydispersity [31,32,33], the synthesis of carboxyl functionalized microgels with the co-polymerization of acrylic acid into the microgel is a straightforward, efficient, and well-controlled procedure. In this work, we showed that these carboxyl functionalized microgels can be efficiently converted to amine functionalized microgels by the coupling of diamines to the carboxyl groups, which offers a more efficient yet simple alternative for the preparation of amine functionalized microgels compared to their direct synthesis. Furthermore, the proposed functionalization method is a simple aqueous procedure performed at room temperature, making it a practical and convenient approach for synthesizing primary amine functionalized PNIPAm microgel particles. 

The key parameter of the coupling efficiency is the EDC excess used in the reaction. We found that a 12-fold EDC excess (compared to the carboxyl content of the reaction mixture) was required to maximize the amine functionalization. However, further increase of the EDC excess did not result in further significant increase of the amine functionalization. Under these circumstances, we turned about two thirds of the carboxylic acid content of the microgel into amine functionality, resulting in PNIPAm microgels with 6–7 mol% amine content. While this results in cationic microgel particles with high amine content, the most important limitation of the proposed method is that full conversion of the carboxylic groups to amine groups could not be achieved. Due to the presence of the residual carboxyl groups in the microgel particles, the amine functionalized microgels showed complex and strongly pH dependent swelling and surface charge characteristics, which can be an important tool in the development of responsive systems for future applications. 

Finally, it should be noted that the identification and application of suitable drug carriers is a major challenge in drug delivery [57] to avoid acute toxicity, short circulation time, and limited targeting. The primary amine functionalized microgels bare positive charges under normal physiological conditions (pH ~ 7), where enzymes and several other biologically active molecules such as oligonucleotides are negatively charged. This can facilitate the efficient loading of the amine functionalized microgels with these molecules, implying their potential use to develop efficient delivery vehicles in pharmaceutical applications. Their positive charge also facilitates their interaction with the negatively charged cell membranes and facilitates their cellular uptake. If the microgels were prepared with a biodegradable (e.g., acetal or disulfide) crosslinker, then the controlled release of the microgel load could also be facilitated. In addition, the drug-loaded microgels could also be incorporated into stretchable patches for transdermal drug delivery systems [58] or into inks suitable for the 3D-printing of hydrogel constructs [59]. Thus, we foresee that the primary amine functionalized microgels could be used in a wide range of applications ranging from drug delivery and tissue engineering to complex elastic devices such as flexible sensors.

## 4. Materials and Methods

### 4.1. Materials

*N*-isopropylacrylamide (NIPAm, 99%) was purchased from Acros Organics and was recrystallized from n-hexane. *N*,*N*′-methylenebis(acrylamide) (BA, 99%) was purchased from Alfa Aesar and was recrystallized from methanol. Acrylic acid (AAc, 99+%) was obtained from Sigma Aldrich and was purified by vacuum distillation prior usage. Sodium dodecyl sulfate (SDS, 99%+) was received from Sigma Aldrich and was purified by recrystallization from absolute ethanol. Ammonium persulfate (APS, ACS Grade) was purchased from Amresco VWR and was used as received. *N*-(3-Dimethylaminopropyl)-*N*′-ethylcarbodiimide hydrochloride (EDC, BioXtra), 2-(*N*-Morpholino)ethanesulfonic acid hydrate (MES, BioXtra), and Cadaverine dihydrochloride (CAD, Sigma Aldrich, Burlington, MA, USA) were used as received. All solutions were prepared in ultrapure MilliQ water (Total Organic Content ≤ 5 ppb, Resistivity ≥ 18 MΩcm).

### 4.2. Synthesis of Microgel Particles

Copolymer microgel particles were created by free-radical precipitation polymerization in an aqueous solution using a method developed by McPhee et al. [19] To produce 200 mL dispersion of microgels containing 10 mol% acrylic acid and a crosslink density (*R* = *n_NIPA_*/*n_BA_*) of 70 with a total monomer concentration of 130 mM, the following steps were taken: First, 250 mL of MQ water was placed in a double-walled glass reactor and purged with argon at 80 °C for one hour. After an hour, 77 mL of the purged water was removed from the reactor and stored under argon pressure. The required amount of NIPAm (2867 g) and BA (0.062 g) monomers was measured into a 30 mL glass vial and purged with argon for 15 min before dissolving them in 22 mL of oxygen-free water. AAc (0.375 g/10 mL), APS (0.548 g/10 mL), and SDS (0.375 g/10 mL) stock solutions were prepared in the same manner and stored under argon. Once all materials were dissolved, 20 mL of the NIPAm and BA stock solution and 5 mL of the acrylic acid stock solution were added to the reaction mixture and purged with argon for an additional 15 min. Then, 1 mL of the SDS stock solution was added and purged for another 10 min. To start the polymerization reaction, 1 mL of the APS stock solution was quickly added. The reaction proceeded for 4 h and was stopped by rapidly cooling the mixture and passing oxygen through it.

The resulting microgel dispersion was purified by ultracentrifugation (Beckman Coulter, IN, USA, Optima XPN-100 preparative ultracentrifuge used with a Type 90 Ti rotor and OptiSeal polycarbonate centrifuge tubes). The temperature was set to 25 °C at a speed of 65,000 rpm (362,000× *g*)), alongside decantation and redispersion to remove byproducts such as unreacted monomers and oligomers. Prior to the first centrifugation step, the pH of the dispersion was adjusted to pH 7 by NaOH. After centrifugation, the microgels were redispersed in MilliQ water. The centrifugation process was repeated at least three times, and then the purified microgel dispersion was freeze-dried. To determine the acrylic acid content of the purified microgel, a 1 w% dispersion was prepared, its pH was set to 2 by HCl, and a conductometric acid-base titration was performed by NaOH.

### 4.3. Functionalization of the Microgel Particles by a Diamine Using EDC-Coupling

The EDC-mediated diamine coupling reactions were conducted in 20 mL vials at 25 °C. The microgel concentration was adjusted to yield final carboxyl concentrations of 1 mM or 5 mM in the reaction mixture. In both cases, the EDC excess was set at 1, 3, 6, 12, and 24 times the acrylic acid content of the solutions, respectively. The cadaverine concentration was maintained at 10 times higher than the acrylic acid concentration of the reaction mixture, resulting in 10 mM or 50 mM cadaverine concentrations, respectively. The pH was set at 5.5, and the final MES buffer concentration was 50 mM.

In a typical synthesis, a calculated amount of microgel and water was added to the reaction vessel and thermostated for 30 min at 25 °C. Subsequently, the calculated amount of cadaverine was added, followed by the initiation of the reaction through the subsequent addition of EDC. Samples were regularly collected from the reaction mixture and were purified by centrifugation. The concentration of unreacted EDC in the continuous phase was determined using HPLC-UV.

### 4.4. High Performance Liquid Chromatography (HPLC) Measurements

To follow the monomer conversion during the microgel synthesis and to monitor the changes in the concentrations of EDC during the activation reactions, HPLC measurements were performed. The HPLC system comprised a Jasco UV-2070Plus UV/VIS detector operating at 224 nm and a Jasco PU-4180 RHPLC one-head pump with a flow rate of 1 mL/min (Budapest, Hungary). Samples were injected using a Model RheoDyne 7125 sample injector equipped with a 25 μL sample loop. All samples were separated on a Supelco Analytical Discovery HS C18 reverse phase column (10 cm × 4.6 mm; 5 μm). The eluent consisted of a mixture of 20% ethanol and 80% MiliQ water and the pH was set to 2 with a buffer made of phosphoric acid and sodium dihydrogen phosphate (the ionic strength of the medium was 100 mM) in the case of monomer conversion measurements and a mixture of 30% methanol and 70% MilliQ water containing 15 mM sodium hexane sulfonate when the EDC reaction was monitored. Prior to use, the eluent was filtered through a 1 μm glass filter and degassed under vacuum. The Clarity software package was used for chromatogram analysis.

Samples were regularly taken from the reaction mixture both in the case of the microgel synthesis and in the case of EDC coupling. The microgel particles were separated from the reaction mixture by centrifugation using a Hettich (Vlotho, Germany) 220R centrifuge operating at 48,530× *g* acceleration at 25 °C for 5 min. Finally, samples were taken from the supernatant for the HPLC measurement.

### 4.5. Dynamic Light Scattering (DLS) Measurements

The hydrodynamic diameter of the CAD modified microgel particles was determined using dynamic light scattering measurements. The measurements were obtained using a Brookhaven instrument (Holtsville, NY, USA) consisting of a BI-200SM goniometer and a BI-CrossCor cross-correlation digital autocorrelator. A Coherent (Santa Clara, CA, USA) Genesis MX488-1000 STM monomodal laser emitting vertically polarized light with a wavelength of 488 nm was used as a light source. The correlator utilized 218 logarithmically spaced time channels in the 1 μs–0.1 s correlation time range. The pinhole was set to 100 μm. The temperature of the samples was controlled during the measurements by a Thermo-Haake (Karlsruhe, Germany) Phoenix II P2 thermostat. The scattering angle for all samples was set to 60 degrees. The intensity-intensity autocorrelation functions were obtained and then converted to normalized electric field autocorrelation functions using the Siegert relation. The autocorrelation functions were analyzed using the second order cumulant expansion method. Finally, the apparent hydrodynamic diameter of the particles was calculated using the Stokes-Einstein equation.

The measurements of the hydrodynamic diameter were conducted in dilute solutions with a microgel concentration of 50 ppm. Prior to the measurements at each temperature, the samples were allowed to equilibrate for 30 min. In each case, a minimum of ten parallel measurements were performed. The pH of the samples was set by using specific buffers: hydrochloric acid for pH 2, citrate buffer for pH 3, 4, and 5, phosphate buffer for pH 6, 7, 8, and 11, carbonate buffer for pH 9.2 and 10, and sodium hydroxide solution for pH 12. The appropriate buffer concentrations were calculated to maintain a consistent ionic strength of 10 mM. Before the measurements, all samples were filtered through a 0.8 μm cellulose acetate membrane.

### 4.6. Electrophoretic Mobility Measurements

The electrophoretic mobility of the microgel particles was measured by a Malvern (Malvern, UK) Zetasizer Nano Z instrument. This instrument employs a technique called M3-PALS, which combines laser Doppler velocimetry and phase analysis light scattering (PALS) to calculate the average electrophoretic mobility of the measured sample. Each sample was subjected to three parallel measurements, and the relative standard error of the mean electrophoretic mobility was approximately 5–10%. The measurements were performed at a temperature of 25 ± 0.1 °C on diluted microgel samples with a concentration of 50 ppm. The pH of the samples was adjusted by the same buffers we used for the DLS measurements. All samples had a constant ionic strength of 10 mM. Before the measurements, all samples were filtered through a 0.8 μm cellulose acetate syringe filter.

## Figures and Tables

**Table 1:** The carboxyl and the primary amine content of the functionalized (prepared by using 24-fold EDC excess) as well as the original P(NIPAm-co-10%AAc) microgels determined by electrophoretic mobility measurements [55].

	P(NIPAm-co-10%AAc)	NH_2_-Functionalized MG
$c_{{NH}_{2}}$	-	6.33 mmol NH_2_/g MG
$c_{COOH}$	9.61 mmol COOH/g MG	3.65 mmol COOH/g MG

## Data Availability

The data presented in this study are available on request from the corresponding author.
